# α-Synuclein interacts directly with AP2 and regulates its binding to synaptic membranes

**DOI:** 10.1016/j.jbc.2025.108502

**Published:** 2025-04-09

**Authors:** Karina J. Vargas, Jaqulin N. Wallace, Ian Mooney, David J. Owen, Jennifer R. Morgan

**Affiliations:** 1 Cell Biology Department, University of Pittsburgh, Pittsburgh, Pennsylvania, USA; 2 The Eugene Bell Center for Regenerative Biology and Tissue Engineering, Marine Biological Laboratory, Woods Hole, Massachusetts, USA; 3 Cambridge Institute for Medical Research, University of Cambridge, Cambridge, UK

**Keywords:** α-synuclein, AP2, synaptic vesicle recycling, endocytosis, synapse

## Abstract

**α**-Synuclein mutation and aggregation are associated with several neurodegenerative disorders, including Parkinson's disease, dementia with Lewy bodies, and multiple system atrophy. It is expressed in the presynaptic compartment where it regulates clathrin mediated synaptic vesicle endocytosis. We have shown that **α**-synuclein regulates clathrin lattice size and curvature *in vitro*. However, the molecular mechanism by which this occurs remains unknown. Here, we show a strong colocalization between the heterotetrametric clathrin adaptor protein-2 (AP2) and **α**-synuclein at presynapses. Moreover, we report a direct biochemical interaction between the AP2 core domain and the C-terminal domain of **α**-synuclein. We further show that **α**-synuclein binds to isolated synaptic membranes in an ATP-dependent manner, similar to AP2 and the monomeric adaptor protein, 180 KDa (AP180), suggesting that **α**-synuclein, AP2, and AP180 share a common synaptic membrane binding pathway. In contrast, other endocytic proteins, such as clathrin heavy chain and the large GTPase dynamin-1, bind to synaptic membranes independent of ATP. After immunodepleting **α**-synuclein, we observed a specific reduction in AP2 binding to synaptic membranes, indicating that **α**-synuclein interaction with AP2 is necessary to maintain normal levels of AP2 on synaptic membranes. These findings demonstrate that **α**-synuclein plays a critical role in stabilizing AP2 on synaptic membranes, an event that is required for initiation of clathrin-mediated synaptic vesicle endocytosis.

α-Synuclein is a small presynaptic protein that regulates synaptic vesicle trafficking under physiological conditions and that aberrantly aggregates throughout neurons in Parkinson's disease, dementia with Lewy bodies, and other related synucleinopathies (1, 2, 3, 4). Despite the clear links to several neurodegenerative diseases and several decades of research on this protein, the normal functions of α-synuclein and underlying molecular mechanisms have remained surprisingly enigmatic. In physiological conditions, α-synuclein has been implicated in multiple stages of synaptic vesicle trafficking (3, 4). In mammalian neurons, α-synuclein regulates fusion pore kinetics during synaptic vesicle exocytosis (5, 6), endocytosis kinetics (7, 8, 9), and vesicle re-clustering after endocytosis (10). α-Synuclein also regulates synaptic vesicle clustering *in vitro* and *in vivo*, in cooperation with one of its major interaction partners, synapsin, which is a major synaptic phosphoprotein (11, 12, 13, 14). Thus, the current body of evidence indicates that α-synuclein is a multifunctional protein that regulates several critical steps of synaptic vesicle trafficking, and yet very little is known about how each of these functions is molecularly regulated.

The goal of the current study is to further investigate how α-synuclein regulates synaptic vesicle endocytosis. Our prior work implicated α-synuclein in the early stages of clathrin-mediated synaptic vesicle endocytosis prior to dynamin-mediated vesicle fission (7). Initiation of clathrin-mediated synaptic vesicle endocytosis requires the clathrin adaptor proteins AP180 and AP2, which interact with PI(4,5)P_2_ and recruit clathrin triskelions to the plasma membrane (15, 16, 17). Acute or genetic perturbations of AP180 and/or AP2 at *Drosophila*, *C. elegans*, squid, and lamprey synapses, lead to synaptic vesicle endocytosis deficits, implicating these proteins and clathrin-mediated endocytosis in recycling of synaptic vesicles (18, 19, 20, 21, 22, 23, 24). At mammalian synapses, AP2- and clathrin-dependent endocytosis is necessary for efficient synaptic vesicle recycling (25, 26, 27, 28), but is not essential, due to the activation of other pathways in the absence of AP2 (28). Clathrin-independent but AP2-dependent synaptic vesicle recycling has also been reported (29), as has clathrin/AP2-dependent budding from endosomes during ultrafast and activity-dependent bulk endocytosis (30, 31, 32). Thus, clathrin-mediated endocytosis, and more broadly AP2-dependent budding, appears to be critical for proper synaptic vesicle trafficking across many synapse models and modes of vesicle recycling.

Several lines of evidence implicate α-synuclein in the early stages of clathrin-mediated endocytosis. First, co-immunoprecipitations revealed that α-synuclein interacts with AP180 (7), and proximity labeling techniques identified AP180 and AP2 in proximity to α-synuclein in synapses (33). Second, a genetic interaction between α-synuclein and AP2 in *C. elegans* has been observed such that deletion of AP2 in an α-synuclein transgenic exacerbated phosphoserine 129-rich α-synuclein deposits, which is typically associated with neuropathology (34). Third, synapses in the αβγ-synuclein triple knockout mice exhibit very few clathrin-coated pits and vesicles and do not accumulate clathrin coat proteins at synapses, unlike the dynamin knockouts which accumulate clathrin, AP2, and endophilin at the membrane due to fission defects (7). Fourth, α-synuclein also regulates the size and curvature of clathrin lattices *in vitro* (35) possibly *via* CALM/AP180, which control clathrin-coated vesicle lattice and vesicle curvature in neurons (36). All this evidence suggests that α-synuclein, AP180 and AP2 could participate in a common mechanism, and thus we have hypothesized that a malfunction of α-synuclein could produce a deficit in early stages of clathrin-mediated synaptic vesicle endocytosis affecting synaptic viability. However, the molecular mechanisms by which α-synuclein regulates clathrin-mediated vesicle endocytosis are unclear.

Here, we show that, in addition to the interaction with AP180 that was previously reported (7), α-synuclein binds directly to the core domain of AP2. Furthermore, α-synuclein, AP2, and AP180 are recruited to synaptic membranes with a similar nucleotide dependence that is distinct from clathrin and dynamin. Interestingly, immunodepletion of α-synuclein selectively impaired the binding of AP2 to synaptic membranes. These new findings indicate that α-synuclein binds to AP2 and regulates its binding to synaptic membranes, elucidating a molecular mechanism by which α-synuclein can regulate early stages of clathrin-mediated endocytosis and, more generally, synaptic vesicle recycling.

## Results

### **α**-Synuclein and AP2 are localized at synapses

**Figure 1:**
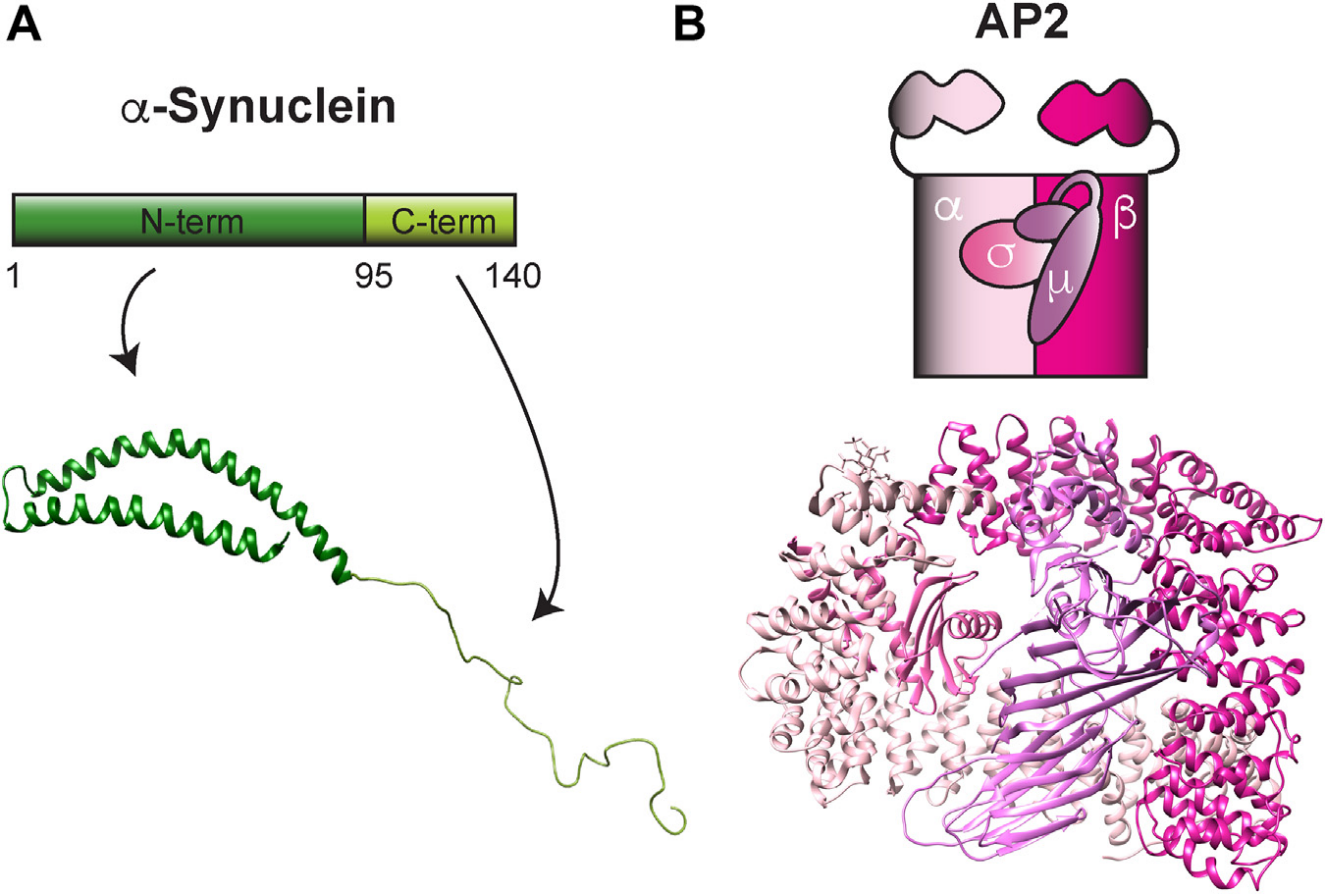
**Structure of α-synuclein and AP2.***A*, (*Top*) diagram of α-synuclein. (*Bottom*) Ribbon model showing the two main domains of α-synuclein from the NMR structure annotated by Ulmer *et al.*, 2005 (UniProt: 1XQ8). *B*, (*Top*) diagram of AP2 showing the α and β adaptins, the larger subunits; and the two smaller subunits μ and σ. (*Bottom*) Ribbon model showing the four subunits of AP2 from X-ray diffraction studies (UniProt: 2VGL). Reference: Collins et al., 2002. AP2 subunits are color-coded to match the diagram above.

**Figure 2:**
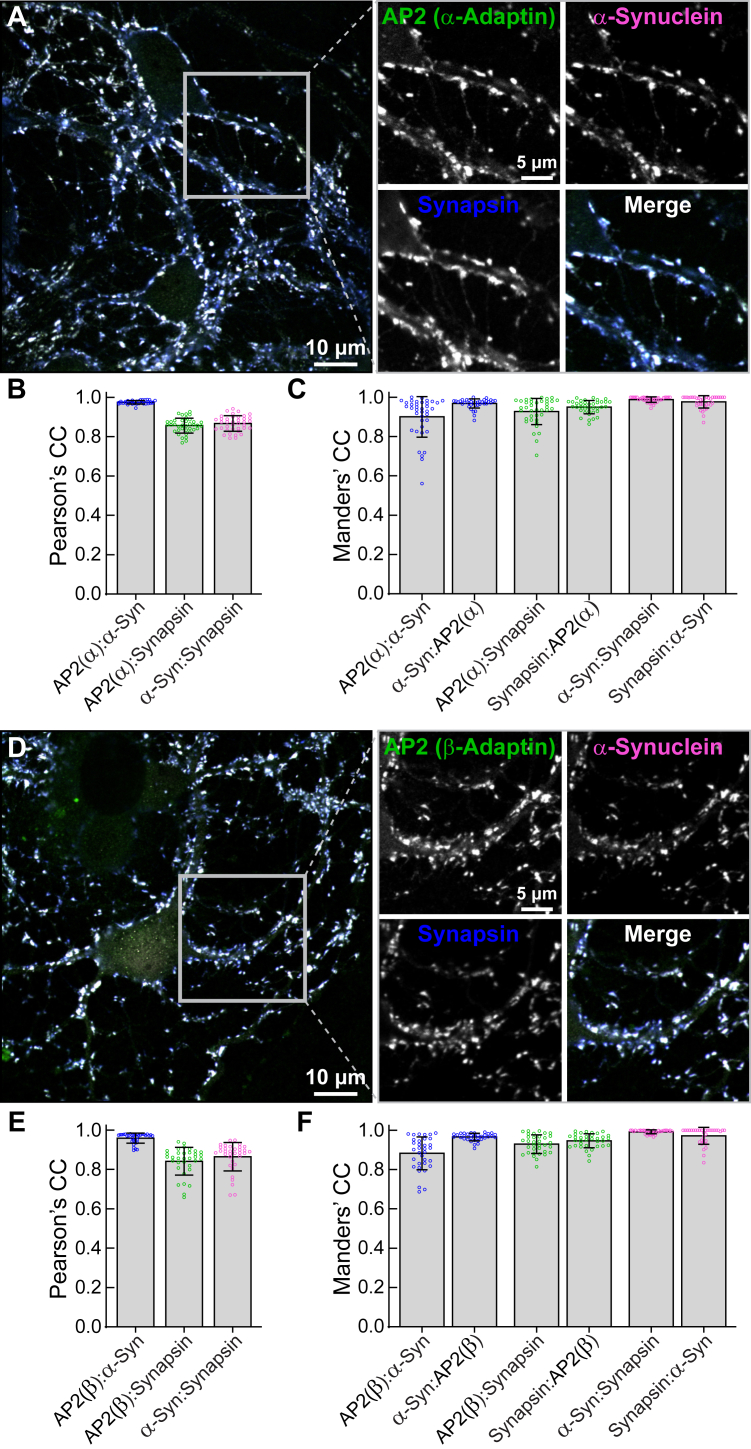
**AP2 subunits α- and β-adaptin colocalize with α-synuclein at presynaptic boutons.***A*, mouse hippocampal neurons (14 DIV) were stained with an α-adaptin antibody (BD Biosciences, Clone 8; 1:500), an α-synuclein antibody (Cell Signaling, clone D37A6; 1:500) and a synapsin antibody (Synaptic systems, SySy 106 004; 1:1000). Insets show areas of high colocalization between α-adaptin and α-synuclein within synapsin positive presynaptic boutons. *B*, Pearson's correlation coefficient (Pearson′s CC) quantification showing strong co-occurrence between these three proteins. *C*, Manders' Co-localization Coefficient (Manders' CC) quantification reveals a strong colocalization between the three channels. *D*, mouse hippocampal neurons (14 DIV) were stained with an β-adaptin antibody (BD Biosciences, Clone 74; 1:500), an α-synuclein antibody (Cell Signaling, clone D37A6; 1:500) and a synapsin antibody (Synaptic systems, SySy 106 004; 1:1000). Insets show areas of high colocalization between β-adaptin and α-synuclein within synapsin positive presynaptic boutons. *E–F*, Pearson′s CC and Manders' CC quantifications reveal a similar correlation between β-adaptin and α-synuclein as was seen with α-adaptin. Data indicate mean ± SD from n = 34 to 38 images, N, 3 independent neuronal cultures.

α-Synuclein is a small, 14 kDa presynaptic protein comprising an N-terminal alpha-helical domain that interacts with membranes (a.a. 1–95) and an intrinsically disordered acidic C-terminal domain (a.a. 96–140) (Fig. 1*A*) (3, 4, 37). Given that α-synuclein regulates early stages of synaptic vesicle endocytosis (7), we set out to determine whether it can interact with the clathrin adaptor AP2, which is involved in synaptic vesicle endocytosis and cargo retrieval across many synapse models (20, 24, 29). AP2 is a heterotetrameric complex comprising two large subunits, α- and β-adaptin (100–130 kDa), as well as a medium subunit μ2-adaptin (∼50 kDa) and a small subunit σ2-adaptin (17 kDa) (Fig. 1*B*) (38, 39, 40). To begin, we examined their localization patterns in neurons and specifically at synapses. Mouse hippocampal neurons (14 DIV) were prepared and immunostained for three proteins: synapsin, a membrane-associated synaptic vesicle marker we used as a presynaptic bouton label; α-synuclein; and either α- or β-adaptin, the two large subunits of AP2. Previous studies have shown that α-synuclein is highly enriched in presynaptic boutons by its colocalization with integral synaptic vesicle markers (41, 42, 43), which we validated here by its overlap with synapsin (Fig. 2, *A* and *D*). Although expressed more widely throughout neurons, including in the soma, we found that both α- and β-adaptin were also highly enriched in presynaptic boutons, given their overlap with α-synuclein and synapsin (Fig. 2, *A* and *D*).

We then quantified to what extent the two large subunits, α- and β-adaptin, of AP2 colocalized with α-synuclein and synapsin. To do this, we conducted a Pearson's Correlation Coefficient (Pearson's CC) analysis to measure both the co-occurrence, spatial distribution, and correlation of two labeled proteins through a linear relationship, as well as a Manders' Co-localization Coefficient (Manders' CC) analysis to measure the proportional overlap of one protein that colocalizes with a second protein and *vice versa* (44). α-Adaptin was highly enriched at presynapses with considerable overlap with α-synuclein and synapsin (Fig. 2*A*). Pearson's CC analysis revealed a high co-occurrence and correlation between α-adaptin and α-synuclein (Fig. 2*B*). We also found a high, but slightly reduced, co-occurrence and correlation between α-adaptin with synapsin, and α-synuclein with synapsin, suggesting that AP2 and α-synuclein have a stronger colocalization in comparison (Fig. 2*B*) [PCC_AP2(α):α-syn_ = 0.9742 ± 0.0086); PCC_AP2(α):synapsin_ = 0.8558 ± 0.0381; PCC_α-syn:synapsin_ = 0.8662 ± 0.0396; n = 38 images, N = 3 cultures].

Furthermore, we measured strong Manders' CCs between α-adaptin and α-synuclein, and conversely, α-synuclein and α-adaptin, with the former colocalization slightly reduced due to α-adaptin's wide expression throughout neurons including somata (Fig. 2*C*). A similar trend was observed between α-adaptin and synapsin further validating the enrichment of AP2 at presynaptic boutons, while α-synuclein and synapsin's colocalizations were nearly identical (Fig. 2*C*) [MCC_AP2(α):α-syn_ = 0.8998 ± 0.1034; MCC_α-syn:AP2(α)_ = 0.9680 ± 0.0237; MCC_AP2(α):synapsin_ = 0.9274 ± 0.0669; MCC_synapsin:AP2(α)_ = 0.9495 ± 0.0336; MCC_α-syn:synapsin_ = 0.9880 ± 0.0137); MCC_synapsin:α-syn_ = 0.9762 ± 0.0310; n = 38 images, N = 3 cultures]. The other large subunit of AP2, β-adaptin, was similarly enriched at synapses where it was highly colocalized with α-synuclein and synapsin, as measured by Pearson's and Manders' CC (Fig. 2, *D* and *E*) [PCC_AP2(β):α-syn_ = 0.9588 ± 0.0255; PCC_AP2(β):synapsin_ = 0.8416 ± 0.0702; PCC_α-syn:synapsin_ = 0.8641 ± 0.0724; n = 34 images, N = 3 cultures] [MCC_AP2(β): α-syn_ = 0.8825 ± 0.0833; MCC_α-syn:AP2(β)_ = 0.9652 ± 0.0187; MCC_AP2(β):synapsin_ = 0.9290 ± 0.0470; MCC_synapsin:AP2(β)_ = 0.9464 ± 0.0359; MCC_α-syn:synapsin_ = 0.9917 ± 0.0099; MCC_synapsin:α-syn_ = 0.9714 ± 0.0429; n = 34 images, N = 3 cultures]. Taken together, we found that AP2 has a wider distribution in neurons but is highly enriched at synapses where it colocalizes with α-synuclein and synapsin. Because of their co-enrichment at synapses, we hypothesized that α-synuclein and AP2 may be functionally linked.

### **α**-Synuclein directly interacts with the AP2 core region

**Figure 3:**
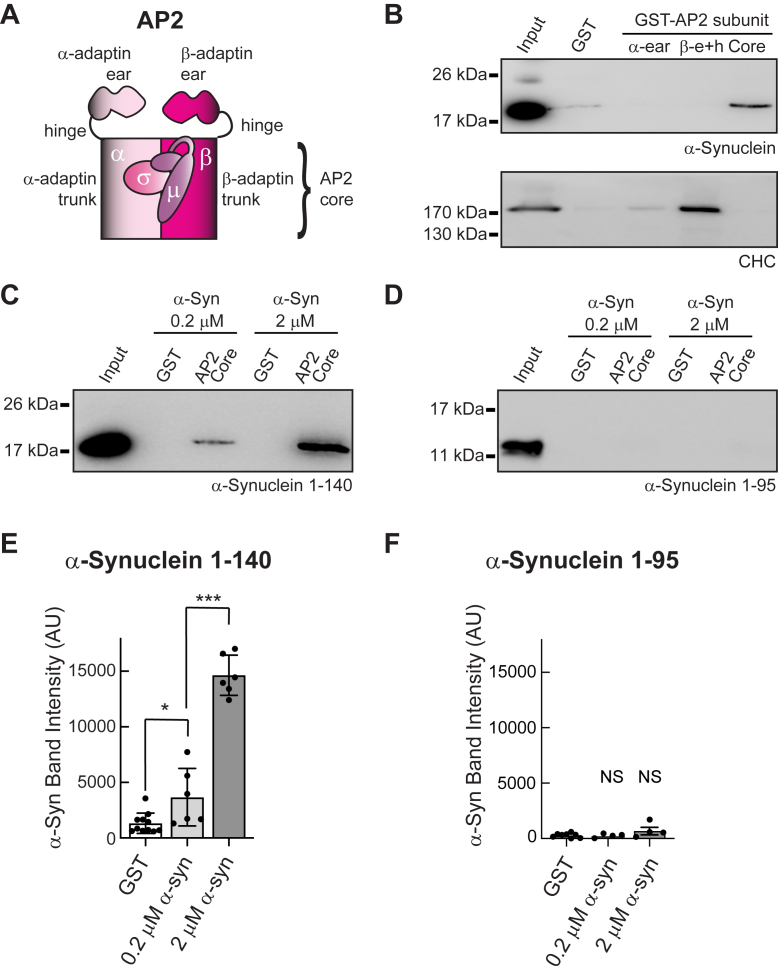
**AP2 and** α**-synuclein interact directly.***A*, diagram of the different AP2 constructs used in the GST pull down experiments. *B*, Western blot showing the GST pull down experiments from post nuclear rat brain homogenate. GST-tagged AP2 Core pulled down α-synuclein from rat brain lysates. α-Synuclein was detected using an anti-pan-synuclein antibody (Abcam 53726; 1:1000). As a positive control, clathrin heavy chain (CHC) was pulled down with the GST-tagged β2-ear plus hinge (BD Biosciences clone23; 1:1000). *C*, Western blot and quantification showing a direct, dose-dependent interaction between GST-tagged AP2 core and full-length α-synuclein (a.a. 1–140). α-Synuclein was detected using Abcam 53726 (1:1000). *D*, in comparison, Western blot and quantification showing that AP2 does not interact with the N-terminal domain of α-synuclein alone (a.a. 1–95), implicating the C-terminus in the interaction. *E–F*, quantification of the interaction between AP2 Core and α**-**synuclein. AU, arbitrary units. Data indicate mean ± SD from n = 6 independent experiments. Statistics: One-way ANOVA, Tukey *post hoc*, *p* < 0.05∗ and *p* < 0.0001∗∗∗. NS indicates “not significant.”

To determine whether α-synuclein and AP2 can interact biochemically, we tested for pulldown of α-synuclein from rat brain protein lysates using GST-tagged fusions of several AP2 domains, including α-adaptin ear, β2-adaptin ear + hinge, and the AP2 core comprising α/β-adaptin trunks, μ2, and σ2 (Fig. 3*A*) (38). Under these conditions, α-synuclein bound weakly but selectively to the AP2 core, but not to α-ear or β2-ear + hinge domains (Fig. 3*B*, top). Binding of clathrin heavy chain (CHC) to α-ear and β2-ear + hinge domains confirmed proper folding of these recombinant proteins (Fig. 3*B*, bottom), as demonstrated in prior biochemical and structural studies (38, 40, 45, 46, 47). To determine whether α-synuclein and AP2 core bind *via* a direct interaction, we repeated the pulldowns using recombinant GST-tagged AP2 core and recombinant full-length human α-synuclein (rPeptide, Inc.). Under these reduced conditions, AP2 core pulled down α-synuclein in a concentration-dependent manner, demonstrating a direct interaction (Fig. 3, *C* and *E*) [GST:1325 ± 918 AU; α-Syn 0.2 μM: 3673 ± 2591 AU; α-Syn 2 μM: 14,641 ± 1813 AU; n = 6; *p* < 0.05∗ and *p* < 0.0001∗∗∗ one-way ANOVA]. To map the interaction, we repeated these experiments using the N-terminal domain (a.a. 1–95) of human α-synuclein and found that the interaction with AP2 core was abolished (Fig. 3, *D* and *F*) [GST: 282 ± 196 AU; α-Syn 0.2 μM: 262 ± 183 AU; α-Syn 2 μM: 736 ± 670 AU; n = 4; not significant, one-way ANOVA]. Thus, α-synuclein directly interacts with the AP2 core complex, likely through its C-terminal domain (a.a. 96–140).

### **α**-Synuclein is recruited to synaptic membranes in a manner similar to AP2 and AP180

**Figure 4:**
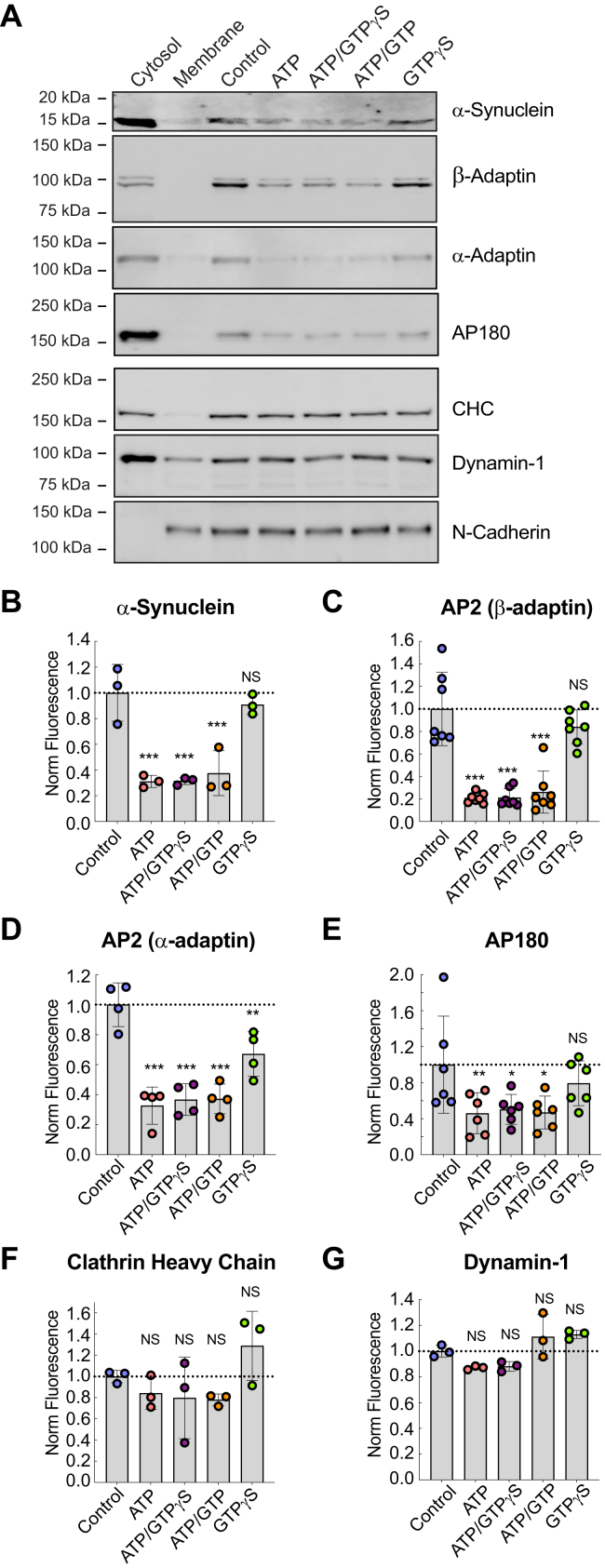
**α-Synuclein, AP2, and AP180 are recruited to the membrane in a nucleotide-dependent manner.***A*, Western blots showing the binding of select endocytic proteins from whole brain cytosol to stripped synaptic membranes in the presence of varying nucleotide conditions. “Cytosol” indicates the input material of soluble proteins. “Membrane” indicates the isolated synaptic membranes. “Control” indicates the soluble proteins that bound to membranes in the absence of nucleotides. α-Synuclein, AP2 (β-Adaptin, α-Adaptin) and AP180 show a similar membrane binding pattern with strongest binding in control or GTPγS conditions, and reduced binding with ATP. In contrast, Clathrin heavy chain (CHC), and dynamin (Dyn-1) binding to synaptic membranes is insensitive to nucleotide conditions. N-Cadherin, a transmembrane protein, was used as a loading control for the membranes. *B–G*, quantification showing the nucleotide-dependence of α-synuclein, AP2, AP180, CHC and dynamin binding to synaptic membranes. Bars indicate mean ± SD for n = 3 to 6 independent experiments. Asterisks indicate statistical significance using One-way ANOVA, Dunnet *post hoc*, ∗ indicates *p* < 0.05; ∗∗ indicates *p* < 0.005; and ∗∗∗ indicates *p* < 0.0005. NS indicates “not significant.”

We next wanted to examine how AP2 and α-synuclein interact with synaptic membranes. We therefore took advantage of an established *in vitro* membrane binding assay, which utilizes purified synaptic membranes isolated from mouse synaptosomes, comprising the full complement of endogenous lipids and integral membrane proteins (48, 49, 50). Isolated synaptic membranes were stripped of associated proteins and incubated with complete mouse brain cytosol in a variety of nucleotide conditions, since a number of endocytic proteins are known to require ATP or GTP to effectively interact with membrane (50, 51, 52, 53). Using this approach, we found that endogenous α-synuclein exhibited the strongest binding to purified synaptic membranes under control, nucleotide-free conditions (Fig. 4*A*). In the presence of ATP, there was weaker binding of α-synuclein to synaptic membranes, while no difference (from control) was observed in the presence of GTPγS alone (Fig. 4, *A* and *B*). A similar pattern of membrane binding was observed for the β-adaptin and α-adaptin subunits of AP2, as well as AP180 (Fig. 4, *A*, *C*–*E*). In contrast, the binding of clathrin heavy chain (CHC) and dynamin-1 to synaptic membranes was unaffected by ATP or GTP nucleotides (Fig. 4, *A*, *F*–*G*). Thus, α-synuclein is recruited to synaptic membranes in a similar manner to the clathrin adaptor proteins AP2 and AP180, which is in line with their established interactions and proposed roles in the early stages of clathrin-mediated endocytosis (7, 35).

### **α**-Synuclein is required for proper recruitment of AP2 to synaptic membranes

**Figure 5:**
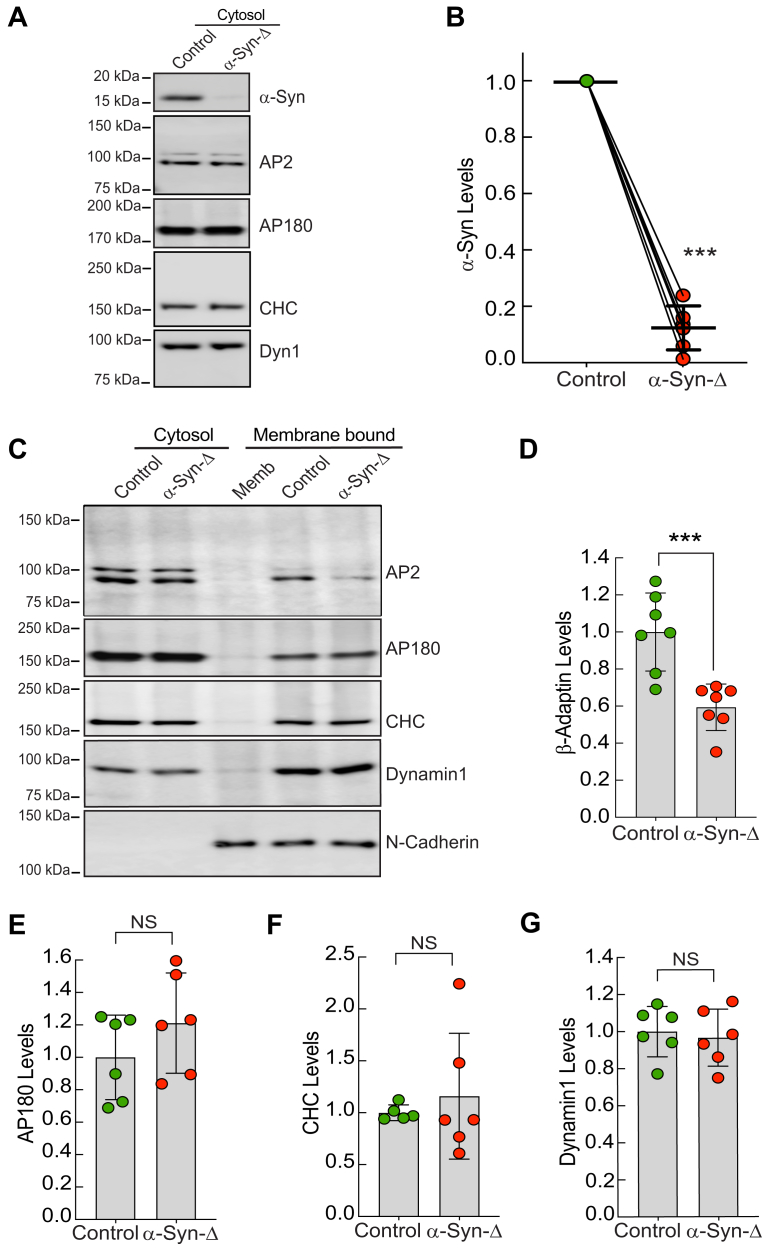
**AP2 recruitment to the synaptic membrane is impaired after acute immunodepletion of α-synuclein from brain cytosol.***A*, Western blot of control and α-synuclein immunodepleted cytosol showing the decrease in the levels of α-synuclein and the unchanged levels of other selected endocytic proteins. *B*, quantification of six independent western blots as in *A* show 88% depletion of α-synuclein compared to control cytosol. Data indicate mean ± SD for n = 6 independent experiments. Statistics: *t* test, *p* < 0.0001∗∗∗. *C*, Western blot showing the binding of selected endocytic proteins to stripped membranes in control and α-synuclein immunodepleted conditions. Only AP2 binding was substantially reduced, as indicated by loss of AP2 signal (clone 100/1, sigma; 1:200). *D–G*, AP2 binding to the membrane was significantly reduced when α-synuclein was immunodepleted from the cytosol. However, AP180, clathrin heavy chain (CHC), and Dynamin-1 remained statistically unchanged. Bars indicate mean and SD from n = 6 to 7 independent experiments. Asterisks indicate significance by Student's *t* test, *p* < 0.0002∗∗∗. NS indicates “not significant.”

Next, we set out to determine whether the presence or absence of α-synuclein might impact the recruitment of AP2 and/or AP180 to synaptic membranes. We therefore immunodepleted α-synuclein from mouse brain cytosol using a monoclonal antibody raised against full-length human α-synuclein (4D6, BioLegend). Immunodepletion of α-synuclein resulted in a robust 88% reduction in the levels of α-synuclein in cytosol samples (Fig. 5, *A* and *B*) [Control: 1.0; α-Syn-Δ: 0.12 ± 0.08; n = 6; *p* < 0.0001; Student's *t* test]. In contrast, AP2, AP180, clathrin heavy chain, and dynamin levels remained unchanged in the α-synuclein-immunodepleted cytosol, confirming the specificity of the immunodepletion for α-synuclein **(**Fig 5*A* and Fig. S1**)**.

We then repeated the membrane binding experiments using the control and α-synuclein-immunodepleted cytosols. After immunodepletion of α-synuclein (α-Syn-Δ), AP2 recruitment to synaptic membranes was significantly reduced by >40% [AP2 β-adaptin: Control = 1.00 ± 0.21, α-Syn-Δ = 0.59 ± 0.13, n = 7; Student's *t* test = 0.0004] (Fig. 5, *C* and *D*). In contrast, the recruitment of other endocytic proteins remained statistically unchanged, including AP180, clathrin heavy chain and dynamin [(AP180: Control = 1.00 ± 0.26, α-Syn-Δ = 1.21 ± 0.31, n = 6; Student's *t* test = 0.1153) (CHC: Control = 1.00 ± 0.08, α-Syn-Δ = 1.16 ± 0.61, n = 5–6; Student's *t* test = 0.4995) (Dynamin: Control = 1.00 ± 0.14, α-Syn-Δ = 0.97 ± 0.15, n = 6; Student's *t* test = 0.3502)] (Fig. 5, *C*, *E*–*G*). Thus, α-synuclein is required for proper recruitment and stabilization of AP2 on synaptic membranes, and this effect is selective for AP2 over other major clathrin coat components.

## Discussion

### **α**-Synuclein promotes or stabilizes AP2 binding to synaptic membranes

**Figure 6:**
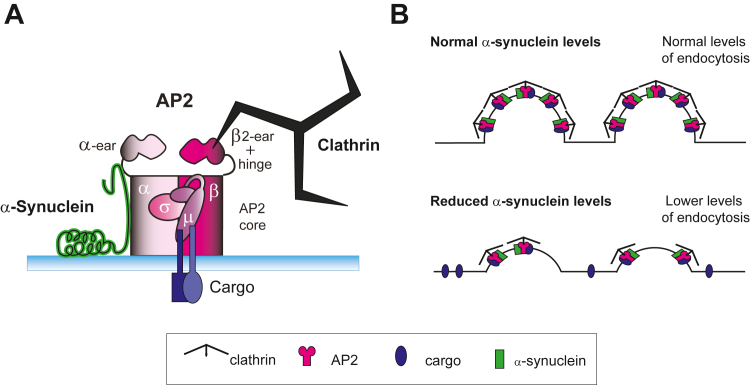
**AP2 binding to synaptic membranes is affected by α-synuclein levels.***A*, this study identified a direct interaction between the core region of clathrin adaptor AP2 and the C-terminal domain of α-synuclein that is required for maintaining proper AP2 levels on synaptic membranes. As α-synuclein and AP2 interact directly and both bind to PI(4,5)P_2_, this could provide an efficient means to facilitate initiation of clathrin coat formation, promote cargo internalization, or some other cellular function during early stages of endocytosis. *B*, reduced α-synuclein levels *via* immunodepletion led to significantly less AP2 on synaptic membranes, and we hypothesize this would interfere with early stages of clathrin-mediated vesicle endocytosis at synapses.

The effective recruitment of initiation factors to the membrane is necessary for the formation of clathrin-coated pits and hence synaptic vesicle endocytosis as well as cargo internalization (54, 55, 56). For clathrin to nucleate on membranes, the primary initiation factors, AP180 and AP2, must bind to the membrane (16, 20, 21, 50, 57, 58). We found that α-synuclein, AP180, and AP2 shared a common pattern of binding to synaptic membranes, implying that they can all attach to similar proteins and/or lipids (Fig. 4). We found that the AP2 complex is enriched at presynapses where it strongly colocalizes with α-synuclein and synapsin (Fig. 2). We observed direct binding between α-synuclein and AP2 (Fig. 3) and that the binding of AP2 to the membrane decreased with immunodepletion of α-synuclein from the cytoplasm (Fig. 5), indicating that their shared pattern of membrane binding is likely due to their interaction (Fig. 6). The impact of α-synuclein on AP2 levels on membranes may be attributed to a cytosolic interaction between α-synuclein and AP2. This could lead to alterations in the conformation of AP2, which may promote AP2 trafficking to the membrane. An alternative explanation could be that α-synuclein stabilizes “AP2 bound to synaptic membranes” conformational state. As both of these possibilities are plausible, we will pursue this question further in our future research.

### **α**-Synuclein interacts directly with the AP2 complex

In this study, we found for the first time a direct protein-protein interaction between α-synuclein and AP2 in brain tissue (Fig. 3). We demonstrated that α-synuclein binds to the core of the AP2 complex. The core of the AP2 complex interacts in the membrane with PI(4,5)P_2_, cargo proteins, and the FCHO interdomain linker, which is a nucleator of clathrin-mediated endocytosis. (17, 40, 54, 59, 60). Our data show that the AP2 core binds to the C-terminal domain of α-synuclein (a.a. 96–140), which is the region on α-synuclein that binds to the synaptic vesicle v-SNARE protein, VAMP2 (43, 61). This presents the interesting possibility that during the transition from exocytosis to endocytosis, α-synuclein experiences a “switch” from interacting with VAMP2 to AP2. Furthermore, α-synuclein attaches to the membranes *via* its N-terminal, which permits α-synuclein to stay attached to the membranes while leaving its C-terminal free to attach to the AP2 core (Fig. 6*A*). Going forward, it will be important to further map the amino acid residues within α-synuclein that bind to AP2, as well as the impacts of critical post-translational modifications within the C-terminal domain, including phosphorylation of serine 129 (pS129) that is known to modulate α-synuclein function in both physiologic and diseased conditions (49, 62, 63). Several biochemical studies report that pS129 α-synuclein binds more strongly to synaptic membranes (49), as well as a number of proteins involved in exocytosis and endocytosis, including AP2, compared to wild-type α-synuclein (62, 64). Understanding how these and other C-terminal modifications impact the interaction between α-synuclein and AP2, as well as any downstream effects on their membrane binding, clathrin coat formation, and synaptic vesicle endocytosis, will be important next steps in defining the multifaceted roles of α-synuclein at synapses.

Supporting our current findings, other studies have shown indirectly that α-synuclein and AP2 may interact. Those studies include a proximity labeling of α-synuclein in the synapse (33), a genetic interaction in *C. elegans* (34), and a GST pull-down with an α-synuclein peptide (64). Importantly, we found that the interaction between α-synuclein and AP2 is direct, bringing up important implications for how they work together on the plasma membrane in neurons. It is possible that this interaction is a requirement for the adequate binding of AP2 to the plasma membrane, thereby aiding the formation of clathrin-coated pits. The outcome of the interaction between α-synuclein and AP2 in the synapse may be synergy to bind to the membranes or the cargo proteins. It is also possible that α-synuclein competes with membranes or cargo for AP2, indicating an additional degree of regulation for both AP2 membrane binding and the recruitment of cargo to the pits.

### Possible impacts of altered **α**-synuclein and AP2 levels on synaptic vesicle endocytosis

We previously reported that α-synuclein regulates synaptic vesicle endocytosis and affects clathrin lattice size and curvature *in vitro* (7, 35). In alignment with the prior studies, our new results show that α-synuclein interacts with and is necessary for the binding of AP2, the main clathrin adaptor, to the membrane (Fig. 6). We demonstrated that α-synuclein interacts specifically with the core of AP2 and this interaction may be important for the α-synuclein dependent recruitment of AP2 to the membrane. AP2 is now known to be involved in at least two modes of synaptic vesicle recycling: clathrin-mediated endocytosis and AP2-dependent/clathrin-independent synaptic vesicle recycling. Thus, alterations in AP2 recruitment to synaptic membranes, due to changes in α-synuclein levels, could have significant effects on SV endocytosis by inhibiting initiation and maturation of clathrin-coated pits (Fig. 6*B*). In support of this idea, synapses from αβγ−synuclein triple knockout mice exhibited a reduction in SV endocytosis with slower kinetics (7). Conversely, acutely increasing α-synuclein levels at stimulated lamprey synapses resulted in a loss of synaptic vesicles and deep evaginations of the plasma membrane reminiscent of AP2 perturbations (23, 65, 66). Recently, the colocalization of α-synuclein with phosphorylated AP2 (the activated version of AP2) has been shown in cell lines (67), adding to the evidence of a functional interaction between α-synuclein and AP2. In our future research, we aim to discover how the interaction between AP2 and α-synuclein affects the endocytic events that occur after AP2 recruitment to the membrane during the initiation of clathrin-mediated endocytosis, including effects on AP2 conformation and cargo binding (Fig. 6*B*).

Regardless of the fundamental cellular processes where α-synuclein participates, the interaction between α-synuclein and AP2 must be important in physiological conditions as well as in pathological conditions, where α-synuclein is affected. When α-synuclein is not functioning properly, like in the case of familial PD point mutations or lack of function phenotypes, there may be less AP2 available on the membrane, which would decrease the rate of synaptic vesicle endocytosis. This deficit in neurotransmission eventually would lead to neurodegeneration. Going forward, it will be important to establish the precise impacts of altering α-synuclein or AP2 levels on synaptic vesicle endocytosis, as any perturbation of the α-synuclein-AP2 interaction is likely to have profound impacts on synaptic function.

## Experimental procedures

### Animal use

All animal procedures were approved by the Institutional Animal Care and Use Committees at the Marine Biological Laboratory in Woods Hole, Massachusetts (IACUC #20–21) and the University of Pittsburgh (IACUC # 24013143) in accordance with standards set by the National Institutes of Health. For neuron culturing, we housed a pregnant mouse (*Mus musculus* C57BL/6*;* female) obtained from The Jackson Laboratory (Bar Harbor, ME) until delivery. Mouse postnatal day 1 (*M. musculus* C57BL/6*; males and* females) were euthanized by decapitation, and hippocampi were isolated for the neuronal culture while the female mouse was sacrificed using Isoflurane (Forane). For the membrane recruitment assays, we obtained adult mice (*M. musculus* C57BL/6*;* males and females) from The Jackson Laboratory (Bar Harbor, ME) and housed them for 1 to 2 days at the Marine Biological Laboratory animal facility until they were euthanized by Fluriso and the brains harvested for purification of synaptosomes membranes and cytosolic proteins. For biochemical GST pull-down experiments, we purchased frozen adult rat (*Rattus norvegicus*) from BioChemed Services, Inc.

### Immunofluorescence of hippocampal neurons

Mouse hippocampal neurons were cultured as described previously (68). In brief, hippocampi were isolated from the brains of mice postnatal day 1, incubated in 2% papain, and mechanically disaggregated. After counting cells, 50,000 cells were cultured on coverslips with a layer of Matrigel. The neurons were maintained in 2% B27, 2 μM AraC MEM for 14 days *in vitro*. They were fixed in 4%PFA/4%sucrose in 0.1 M PBS pH 7.4, blocked with 4% BSA and permeabilized using 0.1% Triton X-100. We stained the neurons overnight using primary antibodies against AP2 (clone 74 β−adaptin, mouse monoclonal, BD Biosciences; 1:500 and clone 8 α−adaptin, mouse monoclonal, BD Biosciences; 1:500), α-synuclein (clone D37A6, rabbit polyclonal, Cell Signaling; 1:500) and synapsin1/2 (Synaptic systems, SySy 106 004; 1:1000). We used Goat anti-Mouse IgG (H+L) Cross-Adsorbed Secondary Antibody, Alexa Fluor 488 for the AP2 subunits, Donkey anti-Rabbit IgG (H+L) Highly Cross-Adsorbed Secondary Antibody, Alexa Fluor 555 for α-synuclein, and Goat anti-Guinea Pig IgG (H+L) Highly Cross-Adsorbed Secondary Antibody, Alexa Fluor 633 for synapsin1/2. As mounting media, we used Invitrogen ProLong Gold antifade reagent. Images were obtained at room temp using the Apo TIRF 60x Oil DIC N2/Aperture: 1.4 on a Nikon Ti2 AX with the Nikon NSPARC detector, running the software Nikon NIS Elements AR Version 6.10.01. To determine the colocalization of the two large subunits of AP2, α- and β-adaptin, with α-synuclein and synapsin1/2, we analyzed the overlap of fluorescence associated with each channel using two established coefficients, Pearson's Correlation Coefficient (PCC) and Manders' Co-localization Coefficient (MCC) (44). An automatic thresholding algorithm was used to determine the background as previously described (69). In brief, this was achieved by iteratively dropping the threshold for the second channel (the farther red-shifted fluorophore) until the PCC for the image crossed zero. The first channel's threshold is related to the second channel's threshold through a linear least-squares fit of the first *versus* second channel's intensity data. PCC and MCC values were calculated using code written in MATLAB (https://github.com/jaqulinwallace/MCC_PCC_MATLAB_Code) and verified in the FIJI plugin JACoP. Data were analyzed from n = 34 to 38 images from N = 3 independent neuronal cultures. GraphPad Prism 10.4.1 was used for all graphical and statistical analysis, and all values reported in graphs indicate mean ± standard deviation (SD).

### GST-protein purification and pulldowns

GST fusions of several AP2 complex components (*i.e.* AP2 core, α-ear, and β-ear plus hinge) were expressed in BL21 *E. coli* and purified, as previously described (17, 38). To generate the rat brain lysates for the GST pulldowns, one frozen rat brain was homogenized with five volumes (9 ml) of ice-cold pulldown buffer (250 mM NaCl, 10 mM Tris pH8.7, 2 mM DTT) containing protease inhibitors (cOmplete, Pierce), and centrifuged at 1000*g* for 10 min at 4 ^°^C. The supernatant was collected, and Triton X-100 was added to a final concentration of 1%, followed by overnight incubation while rotating at 4 ^°^C. Next, the detergent-solubilized homogenate was centrifuged at 100,000*g* for 30 min at 4 ^°^C. Finally, the supernatant was collected, after which the total protein concentration in the rat brain lysates was measured using a BCA assay. Standard GST pulldowns were performed using 100 μg of recombinant GST fusion protein and 5 mg of rat brain protein lysates. After 3 h of incubation with constant rotation, the glutathione Sepharose beads were washed 3 × 15 min in pulldown buffer with 500 mM NaCl. Isolated pull-down proteins of interest were eluted from the beads and detected using standard Western blotting and enhanced chemiluminescence. For the protein-protein interaction, 100 μg of recombinant GST fusion protein with 0.2 μM and 2 μM of recombinant synuclein were incubated in a final volume of 500 μl using pulldown buffer. After 2 h of rotation at 4 ^°^C, beads were washed 3 × 15 min in pulldown buffer with 500 mM NaCl. The interacting proteins were eluded from the beads and detected using standard Western blotting and enhanced chemiluminescence.

### Membrane binding assays

Membrane binding assays were performed as previously described (48, 49). Crude synaptosomes were purified from frozen mouse brains (BioChemed Services, Inc.) using a discontinuous sucrose gradient, from the bottom to the top: 0.65 M, 0.85 M, 1 M, and 1.2 M sucrose. Synaptosomes were collected from the 1/1.2 M interface. Pure synaptosomes were washed, pelleted, and resuspended in 4 ml ice-cold deionized water for hypotonic lysis. Then, HEPES pH 7.4 was added to reach a final HEPES concentration of 7.5 mM, and the membranes were isolated by high-speed centrifugation (100,000*g* for 20 min). Later, they were stripped of associated proteins by incubating the membranes in 0.1 M Na_2_CO_3_, 15 min at 37 °C. Then, the membranes were washed in and resuspended in 2 ml of cytosolic buffer (25 mM HEPES-NaOH, pH 7.4, 120 mM potassium glutamate, 2.5 mM magnesium acetate, 20 mM KCl, and 5 mM EGTA-NaOH, pH 8.0, filtered and stored at 4 °C). Proteins were quantified using BCA. Mini cOmplete protease inhibitors (Roche) were added, and aliquots of purified membrane resuspension were flash-frozen and stored at −80 °C until use.

Cytosolic proteins were made from 2 mouse brains. Brains were first washed using 1xPBS and then homogenized in 25 mM Tris-HCl, 500 mM KCl, 250 mM Sucrose, 2 mM EGTA) in a potter homogenizer. The homogenate was centrifuged at 160,000*g* for 2 h at 4 °C and the supernatant was desalted in a GE column using 3.5 ml cytosolic buffer. After measuring protein concentration and adding protease inhibitors, 100 μl aliquots were flash-frozen and stored at −80 °C until use.

For the membrane binding assays, 200 μg of synaptic membranes were mixed with 250 μg brain cytosol proteins in cytosolic buffer and incubated for 15 min at 37 °C. The samples were centrifuged at 100,000*g* for 30 min at 4 °C, washed, and resuspended in 90 μl of cytosolic buffer. After Western blotting using specific antibodies against endocytic proteins, we scanned the membranes in an Amersham Imager 600. The levels of different proteins recruited to synaptic membranes were quantified using Licor Image Studio Lite. Membrane binding data were analyzed from n = 3 to 6 independent replicates for all experiments shown. No outliers were identified in any of the datasets using the ROUT method, and all datasets shown passed the D'Agostino and Pearson normality test (α = 0.05; *p* > 0.05). GraphPad Prism was used for all graphing and statistical analyses.

### SDS-PAGE and western blotting

For both GST pulldowns and membrane binding assays, samples were loaded onto 6% or 12% SDS-PAGE gels. After electrophoresis, the proteins were transferred onto PVDF membranes. Western blots were performed using standard procedures. After transfer, the PVDF membranes used for α-synuclein immunoblots were fixed lightly with 0.4% paraformaldehyde in PBS for 15 min, washed 3 times with PBS, and blocked in TBS buffer (10 mM Tris pH 7.6, 150 mM NaCl) with 5% nonfat dry milk and 5% goat serum. Then, the membranes were incubated overnight with primary antibodies: α-synuclein (1:1000), clone 4D6 from BioLegend; beta adaptin (β1/β2 of AP2) (1:200), clone 100/1 from Sigma; AP180 clone 34 (1:1000), dynamin clone 41 (1:1000) and clathrin heavy chain clone 23 (1:1000) from BD Biosciences; and N-Cadherin (1:1000) GTX127345 from GeneTex. After washing in TBST (0.05% Tween-20 1xTBS), the blots were incubated for 2 h with goat anti-rabbit or mouse IgG (H+L) conjugated to Alexa633 (Thermo Scientific) at 1:2000 in blocking solution. Membranes were imaged using the Amersham Imager 600 scanner and quantified using Licor Image Studio Lite.

### Immunodepletion

To immunodeplete α-synuclein from the cytosol, approximately 1.5 mg of brain cytosolic protein sample was incubated with 10 μl of a specific antibody against α-synuclein (1 mg/ml, clone 4D6, BioLegend) for 2 h at 4 °C with constant rotation. The antibody-α-synuclein complex was then incubated with Protein A Sepharose beads (GE Life) for 2 h at 4 °C with constant rotation. The cytosol was then immunodepleted of α-synuclein by sedimenting the beads attached to the antibody-α-synuclein complex. Then, the α-synuclein immunodepleted cytosol (supernatant) was transferred to a new tube for the membrane binding experiments. The amount of α-synuclein immunodepletion was determined in n = 6 to 7 independent experiments by measuring how much α-synuclein remained in the cytosol compared to a complete set of proteins in the cytosol before immunodepletion or the control sample using band intensity analysis from Western blots.

## Data availability

All data are presented in the manuscript.

## Supporting information

This article contains supporting information.

## Conflict of interest

The authors declare that they have no conflicts of interest with the content of this article.
