# Supplementary material for: α-Synuclein interacts directly with AP2 and regulates its binding to synaptic membranes
Source: J Biol Chem. 2025 Apr 9;301(5):108502. doi: 10.1016/j.jbc.2025.108502 (PMC12143788; doi:10.1016/j.jbc.2025.108502)
Supplement: Figure S1 [file mmc1.docx]

**Supporting Information Figure 1. Acute depletion of α-synuclein from brain cytosol does not affect the content of selected endocytic proteins. A-D**. Quantification for 4-6 independent experiments showing mean +/- SD. Bands intensities were measured from western blots, as in Figure 4A-B. Control and of α-synuclein immunodepleted cytosol showing no significant changes of in the levels of selected endocytic proteins: AP2, AP180, CHC and Dynamin-1. Statistics: T-test, NS indicates “not significant.”
