# Supplementary figures and images for: α-Synuclein interacts directly with AP2 and regulates its binding to synaptic membranes
Source: J Biol Chem. 2025 Apr 9;301(5):108502. doi: 10.1016/j.jbc.2025.108502 (PMC12143788; doi:10.1016/j.jbc.2025.108502)

**A**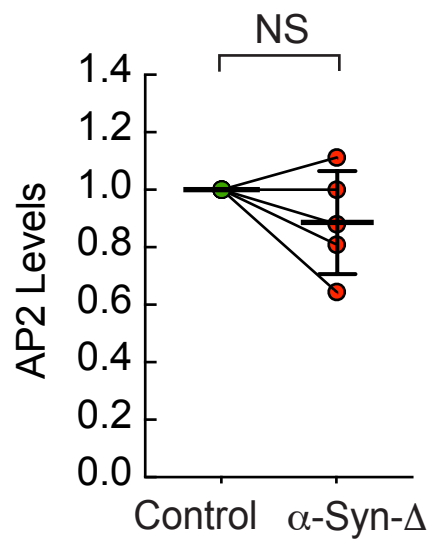**B**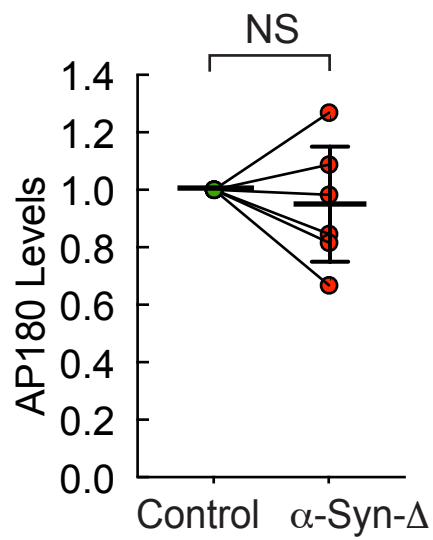**C**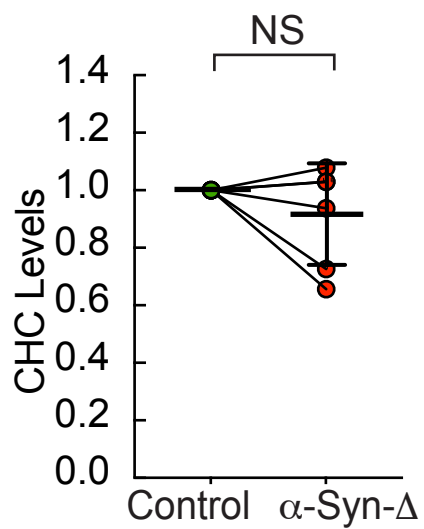**D**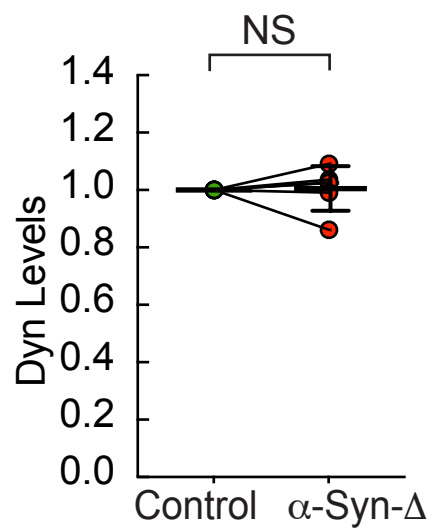

**Supporting Information Figure 1**

Supplement: Supplementary 1 [file mmc2.pdf]
